# Supplementary material for: Cuban Policosanol (Raydel®) Potently Protects the Liver, Ovary, and Testis with an Improvement in Dyslipidemia in Hyperlipidemic Zebrafish: A Comparative Study with Three Chinese Policosanols
Source: Molecules. 2023 Sep 14;28(18):6609. doi: 10.3390/molecules28186609 (PMC10534397; doi:10.3390/molecules28186609)
Supplement: Supplementary file 1 [file molecules-28-06609-s001.zip › molecules-2567212-supplementary.pdf]

Supplementary material

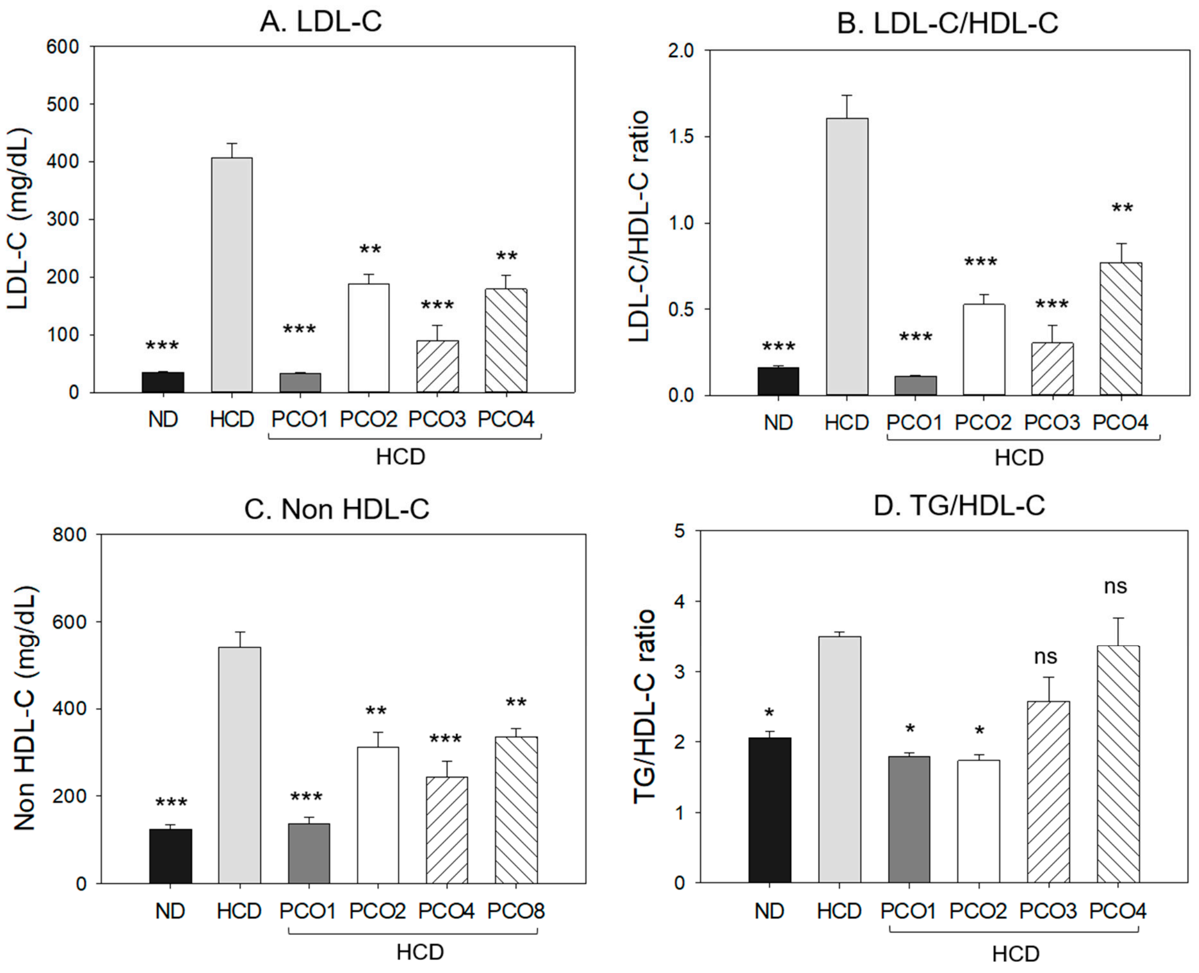

Supplementary Figure S1. Low-density lipoproteins cholesterol (LDL-C) contents, LDL-C/HDL-C ratio, non-HDL-C, and the triglyceride (TG)/HDL-C ratio in blood after 12 weeks supplementation of each policosanol (PCO) under high cholesterol diet consumption. HCD, high cholesterol diet; ND, normal diet; PCO1, Raydel policosanol; PCO2, Xi'an Natural policosanol; PCO3, Xi'an Realin policosanol; PCO4, Shaanxi policosanol. Data are expressed as mean ± SEM. Statistical differences of multiple groups were compared using a one-way analysis of variance (ANOVA) with Dunnett's post-hoc test between the other group and the HCD group. *, $p<0.05$ versus HCD control; **, $p<0.01$ versus HCD control; ***, $p<0.001$ versus HCD control; ns, not significant.
